# The Response of the Pulp-Dentine Complex, PDL, and Bone to Three Calcium Silicate-Based Cements: A Histological Study in an Animal Rat Model

**DOI:** 10.1155/2020/9582165

**Published:** 2020-04-13

**Authors:** Ranjdar Mahmood Talabani, Balkees Taha Garib, Reza Masaeli

**Affiliations:** ^1^Conservative Department, College of Dentistry, University of Sulaimani, Sulaimani-Dream Land B-25, Sulaymaniyah, Kurdistan Region, Iraq; ^2^Oral and Maxillofacial Medicine in Kurdistan Board of Dental Specialties, Department of Oral Diagnosis, College of Dentistry, University of Sulaimani, Sulaymaniyah, Kurdistan Region, Iraq; ^3^Department of Dental Biomaterials, School of Dentistry, Tehran University of Medical Sciences, Tehran, Iran

## Abstract

**Objective:**

The aim of this study was to histologically examine the tissue reaction of three different calcium silicate cements in the closure of perforations in rat incisor teeth. *Material and Methods*. An experimental lateral root perforation with pulp exposure was performed in 32 lower incisors of 16 male Wistar albino rats. They were randomly assigned into three test groups (each including eight teeth) that were filled either by Biodentine (BD) or MicroMega mineral trioxide aggregate (MM-MTA) or EndoSequence root repair material putty (ESRRM putty), besides eight unperforated incisors from the other four rats (control group). The inflammatory response and healing process were evaluated histologically and scored after one and four weeks. Differences among groups were tested by Kruskal–Wallis tests at *P* ≤ 0.05.

**Results:**

In the first week, BD produced more inflammatory response in the pulpal (score 3) than other materials (score 2). Only ESRRM putty showed odontoblast-like cells in 50%, 25% dentine-like deposit, 25% evidence of bone deposition in the drilling site (score 2), and minimum periodontal ligament (PDL) necrosis and disorganization (25%, score 2). After one month, all groups had healthy pulpal tissue, but 25% of ESRRM putty retained score 1 inflammatory response, and 50% of the BD case had an incomplete palisading odontoblast layer (score 3). A thick and regular dentine bridge deposition was seen in the ESRRM putty group in comparison with MM-MTA and BD cases. The cortical plate healing in all ESRRM putty samples was complete (score 3), while an incomplete closure was seen in MM-MTA and BD groups (score 2). Both the MM-MTA and ESRRM putty groups had fully organized PDL (score 2), while in 50% of BD cases, a necrotizing area and disorganized PDL with inflammatory cells infiltration were still present. Statistically significant differences in the scores of any histologic parameters among the three tested materials were observed neither in the 1st nor in the 4th weeks of the experimental period.

**Conclusion:**

Better tissue compatibility and repair of pulpal and periodontal tissue have been detected after lateral perforation in the root of rat incisors when treated with ESRRM putty than MM-MTA and BD. However, the difference was not significant.

## 1. Introduction

Root perforation is a communication between the root canal system and the surrounding tissues through the floor of the pulp chamber or root canal wall of the tooth [1]. It can occur as a result of a large carious lesion in or adjacent to the floor of the pulp chamber, resorptive defects, or iatrogenic factors (i.e., procedural errors or accident) during endodontic treatment [2]. Several factors may interfere with the repair of the defect and, at the same time, affect prognosis [3]. The long-term prognosis of a perforated tooth is dependent upon the location of the perforation, how long the perforation is exposed to oral contamination, and the ability to seal the hole [4]. When a pulp chamber floor perforation occurs, the periodontal ligament (PDL) and the bone tissue are destroyed in variable intensity. The inflammatory process is established, and the risk of the possibility of epithelial proliferation, periodontitis, and progressive alveolar process of bone destruction is increased.

Moreover, if inappropriately treated, perforations could lead to poor prognosis and subsequent tooth loss [5]. Wound healing with reparative mineralized tissue formation represents the optimal end result of treatment; tissue regeneration depends on several host and treatment factors. One of the reasons why perforations tend to have such poor prognosis may be the fact that none of the materials used can accomplish tissue regeneration at the treated site [6, 7]. Calcium silicate-based cement has been considered to be the best choice to seal dentinal defects between the pulp space and the periodontal ligament, as well as a root-end filling after endodontic surgeries [8, 9].

Calcium silicate-based cements have been developed more than 20 years ago with Mineral Trioxide Aggregate as endodontic repair and root-end filling materials [10–12]. Due to their biocompatible properties [13, 14], their clinical usage rapidly expanded towards direct and indirect pulp capping [15], taking into consideration some disadvantages associated with classic MTA formulations such as long setting time, difficulty in handling, poor mechanical properties, and tooth discoloration [10, 16]. Moreover, there is evidence that bismuth oxide used as radiopacifier inhibits cell proliferation [17]. Newer calcium silicate cements have been developed with enhanced physicomechanical and handling properties. MicroMega MTA (MM-MTA; MicroMega Besanchon, France), another formulation of MTA, was developed in 2011 to overcome drawbacks of the original MTA products. It is an injectable osteoconductive, osteoinductive, and biocompatible tricalcium silicate-based cement and also contains calcium carbonate, which helps in reducing the setting time [18].

Biodentine (BD) (Septodont, Saint Maur de Fosses; France) was developed in 2009 as a novel tricalcium silicate-based cement [19]. It was described as a bioactive dentine substitute due to having similar mechanical properties to dentine. Also, it can be used with similar indications to MTA [8, 20]. Following the induction of a moderate inflammatory reaction in rat subcutaneous tissue, BD presents a significant reduction of this response over time, similar to MTA [21]. BD has shown better biological properties than other tricalcium silicate cements such as MTA in in vitro studies in cultures of osteoblasts, periodontal ligaments fibroblasts, and pulp [22, 23]. In addition, BD significantly released more calcium ions with deeper incorporation into the root canal dentine than MTA, hence leading to the formation of a “mineral infiltration zone” [24]. This cement also induces the formation of mineralized tissue sealing furcation perforations in teeth of dogs [25] and dentine bridge formation after pulp exposure of human teeth [8].

An alternative material, EndoSequence Root Repair Material putty (ESRRM putty), was developed as a premixed, injectable material formulated using bioceramic technology. It has been released by Brasseler USA (Savannah, GA) to be used as a clinical replacement for MTA. ESRRM putty has the advantages of faster setting and superior handling properties [26]. Different in vivo studies in rat subcutaneous tissue [27] and in vitro studies on human gingival fibroblast [28] and L929 mouse fibroblasts [29] reported that ESRRM putty showed similar biocompatibility to MTA. Moreover, ESRRM has been found to promote the healing of dental tissue and show similar survival and proliferation of dental pulp cells to MTA using different assays [30]. So, this study aims to investigate and compare the repairing ability of MM-MTA, ESRRM putty, and BD in the modulation of the inflammatory process of pulp, PDL, bone remodeling, odontoblastic differentiation, and dentine bridge formation after lateral perforations in rat incisors. In the null hypotheses, none of the calcium silicate-based materials tested in this study has the ability to repair and/or regenerate pulp, PDL, dentine, and bone when applied on lateral perforations in rat incisors.

## 2. Material and Methods

This study was performed in accordance with the principles of laboratory animal care (NIH publication 85–23, 1985). The national laws on animal use were also observed for the present study by getting authorization from the Ethical Committee for Animal Research of Sulaimani University (no. 9, 6.2.2017).

Sixteen male Wistar albino rats, aged 4–5 months and weighing 250–350 g, with a total of 32 lower incisor teeth, were used in this study. The rats were maintained in individual stainless steel cages under a 12 : 12 light-dark cycle at controlled temperature (21 ± 2C°) and humidity (55 ± 10%), with food and water provided ad libitum, in the animal house at Farabi Comprehensive Center of Excellence in Ophthalmology, Tehran University of Medical Sciences, Qazvin Square, Tehran, Iran.

The rats were anesthetized with an intraperitoneal injection of ketamine 75 mg/Kg of body weight combined with xylazine 10 mg/Kg of body weight. After localizing the site of perforation with a pen marker, an intraoral approach was used to perform the mechanical root perforation of lower rat incisors on the distal border of the labial surface of the root, 5–6 mm below the gingival margin, using a sterile type 2 small-size long-shank carbide round bur on a straight low-speed handpiece with copious saline irrigation. The size of the perforation was standardized to the diameter of the bur head in width (1.5–2-mm) and its depth determined by penetration into the pulp canal. After hemostasis with distilled water and sterile cotton pellet, three calcium silicate-based materials, Biodentine™ (Septodont, St-Maur-des-Fossés, France) (Lot No. 5024200U0), MicroMega MTA (MM-MTA; MicroMega Besanchon, France) (Lot no. 71708614), and EndoSequence Root Repair Material putty (Brasseler USA, Savannah, GA) (Lot no. B19585), were used. The composition of the test materials is shown in Table 1. BD was prepared according to the manufacturer's recommendations and inserted into the perforated site of eight right lower rat incisors with amalgam carrier (Shanghai, China) and condensed using a small-sized ash condenser (Shanghai, China). MM-MTA in an available capsule form was prepared according to the manufacturer's instructions and inserted into the perforations, while ESRRM putty was directly inserted into the holes.

The thirty two lower incisor teeth were divided into 3 experimental groups according to the perforation repair material: BD (*n* = 8/right side) or MM-MTA (*n* = 8/left side) or ESRRM putty (*n* = 8/right side) and one control group (*n* = 8/left side) with no perforation. On the 1^st^ and 4^th^ week after filling the perforations, the animals (*n* = 4 per group/period) were euthanized by carbon dioxide. The mandibles were removed with the perforated root of the lower incisor and its surrounding periodontal tissues immersed in a fixative solution.

**Figure 1:**
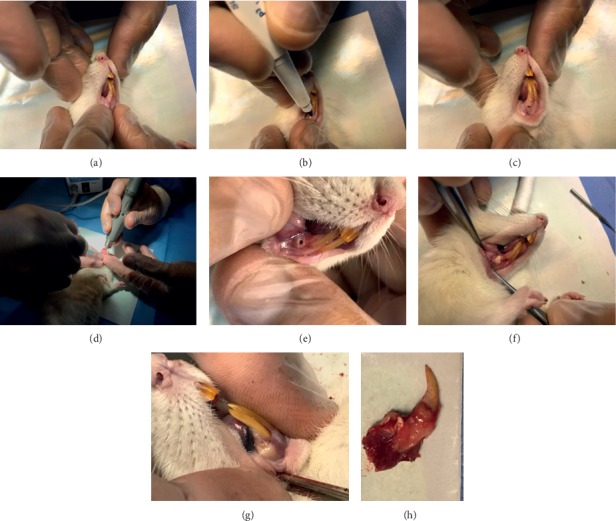
The surgical procedure: (a) lower rat incisor teeth, (b and c) localization of perforation site, (d) surgical perforation procedure, (e) implantation site, (f) material implantation, (g) implantation follow-up evaluation, and (h) excised implantation with surrounding tissues.

The graphically detailed description of the surgery procedure and photographs of the appearance of the implantation site and excision of specimens are shown in Figure 1.

Procedures for the paraffin-embedding and histological evaluation:

The specimens were fixed in 10% buffered formalin solution at pH 7.2 at room temperature for 48 hours. After decalcification for 4–5 weeks in 10% formic acid with 0.5% formaldehyde buffered at pH 7.2, the specimens were dehydrated in graded concentrations of ethanol. Then, they were cleared in xylene and embedded in paraffin. Sections of 5 *μ*m thickness were prepared and stained with hematoxylin and eosin (H&E) for histological studies.

The stained sections were blindly evaluated by two experienced pathologists. Under a light microscope (Olympus, Tokyo, Japan) at ×4, ×10, and ×40 magnifications, all samples were evaluated and scored in terms of pulpal inflammatory response, odontoblast differentiation and dentine bridge formation, calcified bone formation, and continuity and organization of PDL and scored according to criteria modified from Liu et al. [31], Salman et al. [32], and Moreton et al. [33] Table 2.

### 2.1. Statistical Analysis

Statistical analyses were performed using SPSS version 22 software (SPSS, Chicago, IL, USA). The effect of regeneration and repairing on the properties of three calcium silicate-based cements and significant differences between mean were evaluated by using the Kruskal–Wallis test. The level of significance was *P* ≤ 0.05.

## 3. Results

**Figure 2:**
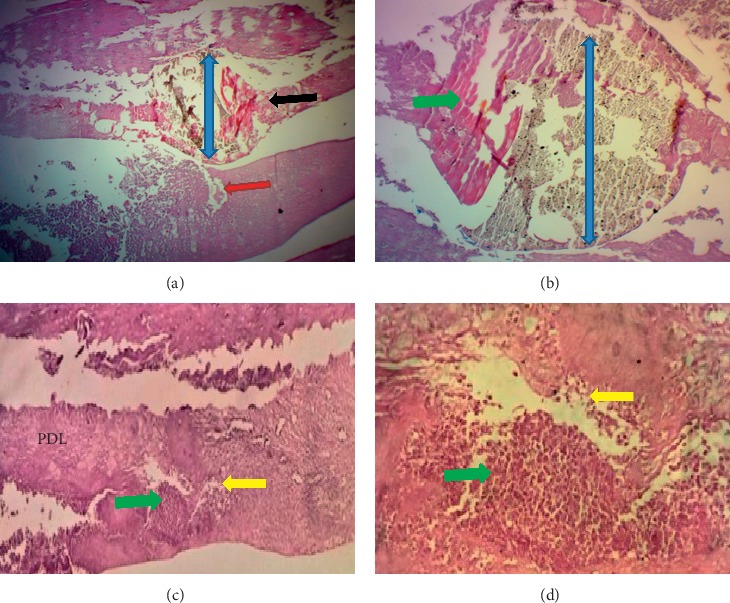
Photomicrograph of an H&E-stained tissue section through the incisor of a rat after 1 week Biodentine (blue up-down arrows) treatment. (a) Longitudinal section showed a gap in dentine (red arrow) and distortion in the pulp (black arrow) (40X). (b) Higher magnification of the necrotic zone (green arrows) in the pulp around BD (100X). (c and d) Severe inflammation (yellow arrows.), localized distortion, and necrosis (green arrow) in the PDL (100X and 400X).

**Figure 3:**
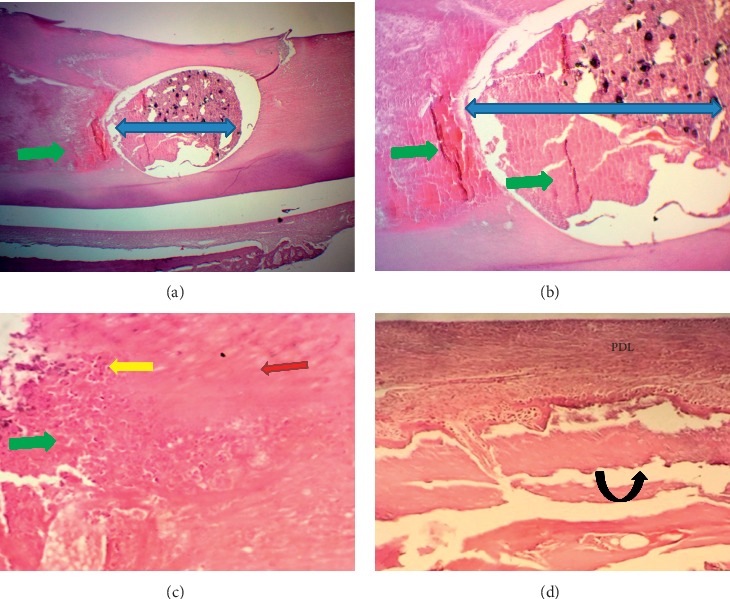
Microphotographs of an H&E-stained longitudinal section for the pulp and periodontium response in the central incisor after 1 week of MM-MTA treatment (blue left-right arrows). (a) (40X) and (b) (100X): evidence of inflammation, necrosis (green arrows) and calcification within the pulp. (c) Irregular dentine (red arrow) deposition (100X). (d) The periodontal ligament had unorganized fibrous tissue and congested capillaries with inflammatory cells infiltration (yellow arrow, 40X). Small woven bone trabeculae (black curved-up arrow) presented at the interface with PDL.

**Figure 4:**
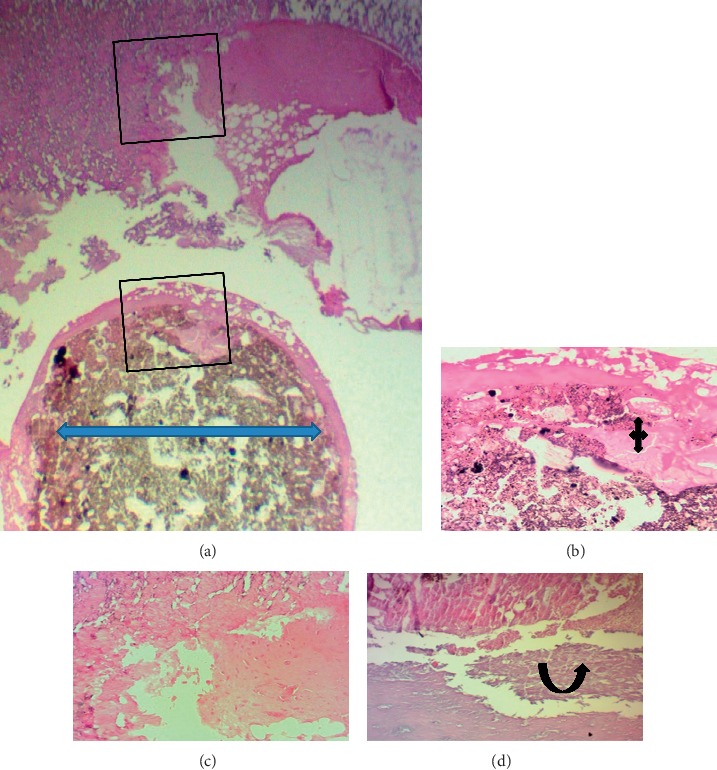
Microphotographs of a longitudinal H&E-stained section through central incisor after one week of ESRRM putty treatment. (a–c) The ESRRM putty (blue left-right arrow) surrounded by and incorporating mineralized deposition pulpal tissue contained a few long dilated and congested blood vessels, and an irregular deposition of reparative dentine (quad arrow) was seen adjacent to multilayers of short odontoblasts ((a) 40X; (b-c) 100X). d: The periodontal ligament had dense fiber bundles running parallel to the root surface and inserted in the newly formed bone with minimum congested capillaries with no inflammatory cells infiltration (100X). The gap in the alveolar bone was filled with irregular and small bone trabeculae (black curved-up arrow).

The results of the mean value scoring for pulp and periodontium response are presented in Table 3. In the first week, sections showed that, in all BD implanted samples, the pulp contained hyperemia, numerous congested capillaries, and an area of necrosis (score 3) (Figure 2). The other tested materials had a mild pulpal response (score 2). They presented moderate hyperemia with less than five congested capillaries per field and high cellularity within a dense fibrous pulpal tissue (Figures 3 and 4).

The drilling procedure and material implantation resulted in odontoblast cell layer destruction (score 0) in all groups (Figures 2 and 3) except in 50% cases in the ESRRM putty group, where a few cuboidal cells were seen adjacent to the implanted materials (score 1) (Figure 4). Within this period, no experimental evidence in the BD group indicated dentine bridge formation (score 0) (Figure 2), and 25% of cases in ESRRM putty and MM-MTA groups showed such deposition (score 1) (Figures 3 and 4).

Gaps in all groups were unclosed; however, signs of material deposits could be seen at the cut edge of the bone in BD and MM-MTA samples (100% score 1) (Figures 2 and 3), with 25% (score 2) of ESRRM putty samples showing evidence of bone deposition (Figure 4). All groups showed a visible area of necrosis and disorganized PDL infiltrated by chronic inflammatory cells (score1). Nevertheless, the ESRRM putty group had nonsignificant necrosis and inflammation (25% at score 2). Seepage of a small increment of ESRRM putty within PDL was devoid of necrosis and surrounded by vital fibrous tissue (Figure 4).

**Figure 5:**
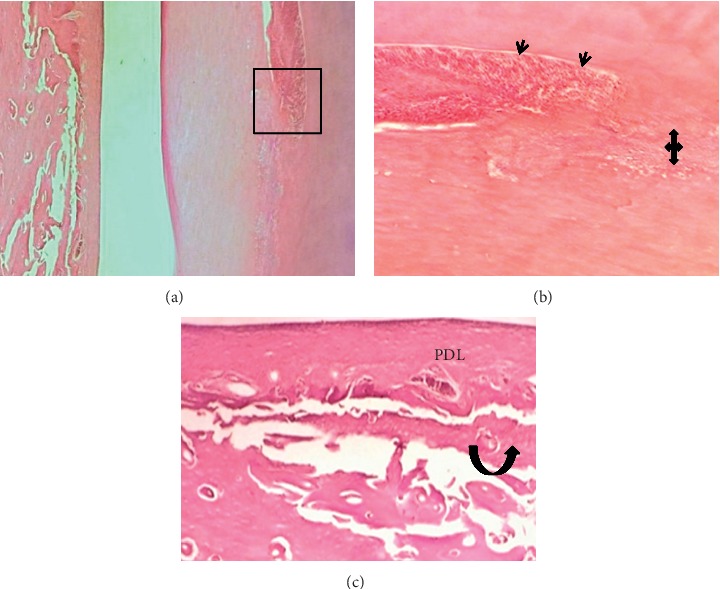
Microphotographs of an H&E-stained longitudinal section through central incisor after 4 wks of Biodentine (BD) treatment. (a-b) A highly cellular pulp tissue obliterated by irregular deposition of reparative dentine (quad arrow) next to multiple layers of small-sized odontoblasts “Black arrow heads” (40X and 100X). (c) The periodontal ligament attached to a thin cementum layer had organized and dense fibrous bundles running parallel to the root surface (principle fibers) besides a few congested and dilated capillaries toward the bone side. There is no inflammatory cells infiltration. The gap in the alveolar bone was filled by multiple small trabeculae and a few vascular marrow tissues were continuous with adjacent bone (black curved up arrow) at the interface with PDL (200X).

**Figure 6:**
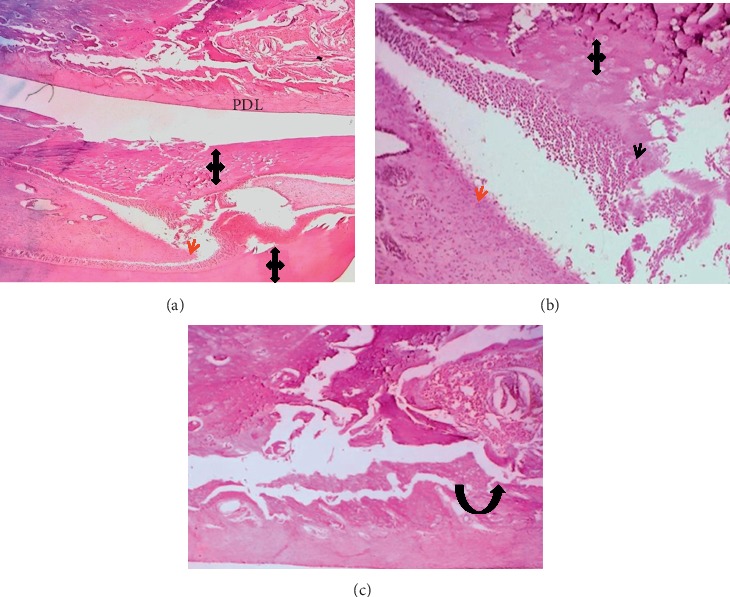
Microphotographs of longitudinal H&E-stained sections through central incisor after 4 wks of MM-MTA treatment. (a-b) The pulpal tissue (red arrow heads) was highly cellular and contained a few dilated and congested blood vessels with no inflammatory cells infiltration and at the experimental site was lined by an area of reparative dentine deposition (quad arrows) with multilayers of short odontoblasts (black arrow head) ((a) 40X, (b) 100X). (C) The periodontal ligament had organized highly dense fibrous bundles running parallel to the root surface (principle fibers) and inserted in the newly formed bone (black curved-up arrow) with minimum congested capillaries and no inflammatory cells infiltration (100X). The gap in the alveolar bone was filled by multiple small trabeculae (black curved-up arrow) and large vascular marrow tissues besides the continuous interface with PDL.

**Figure 7:**
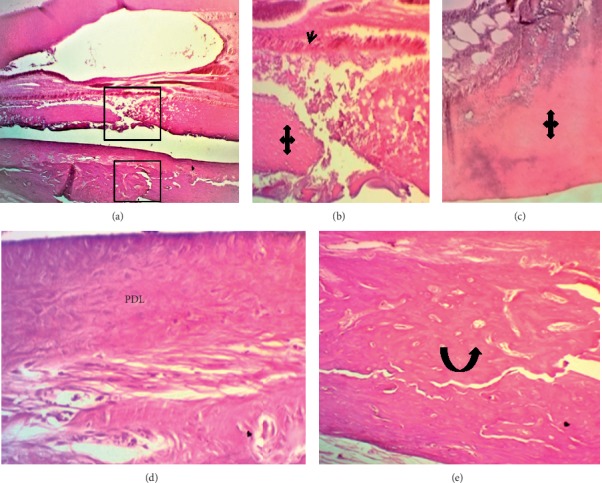
Microphotographs of longitudinal H&E-stained sections through central incisor after 4 wks of ESRRM putty treatment. (a) The pulpal tissue contained a few long dilated and congested blood vessels, and an irregular deposition of reparative (quad arrows) dentine was seen adjacent to multilayers of short odontoblasts (black arrow head) ((a) 40X; (b-c) 100 X). (d) The periodontal ligament (PDL) had organized dense fibrous principle fibers bundles running parallel to the root surface and inserted in the newly formed bone with minimum congested capillaries and no inflammatory cells infiltration (400X). (e) The gap within the alveolar bone (black curved-up arrow) was filled with compact bone and bone marrow (400X).

At the end of the experiment (after one month), the dental pulp in a few cases of ESRRM putty showed less than five congested capillaries per field (25% in score 1). The score for the remaining groups was zero. Both ESRRM putty and MM-MTA groups completely retained the complete palisading pattern of odontoblasts (score 3), while 50% of BD cases showed a partial, incomplete palisading cell pattern (mean score 2.5). The best repair for dentine was presented in the ESRRM putty group, with the results showing thick, regular, and complete dentine formation (score2). Meanwhile, an incomplete and thin irregular dentine bridge was noticed in all cases of the MM-MTA group as well as 75% of BD cases (mean score, 1.3) (Figures 5–7).

Considering the periodontium, all samples of ESRRM putty were associated with calcified bone that completely closed the exposure site (score 3) (Figure 7) in comparison to the partial, incomplete, and noncompact bone deposition extending across less than 1/2 of the drilling site as identified in the BD and MM-MTA groups (score 2) (Figures 5 and 6). Furthermore, both MM-MTA and ESRRMs putty groups had fully organized PDL with no necrosis or inflammation (score 2) (Figures 6 and 7), while in BD (mean score 1.5), 50% of cases showed a necrotizing area with disorganized PDL and inflammatory cells infiltration (Figure 5).

**Figure 8:**
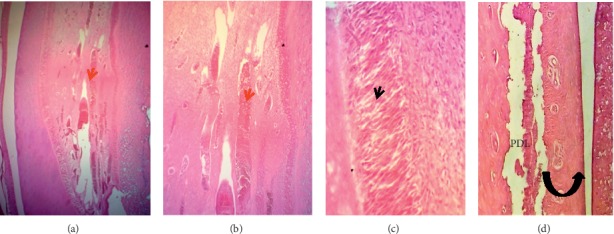
Microphotographs of an H&E-stained longitudinal section through healthy nonperforated lower central incisors (control group). (a–c) The dental pulp (red arrow heads) showed long dilated congested blood vessels, cellular loose connective tissue, and multiple layers of palisaded columnar odontoblast (black arrow heads) cells adjacent to predentine (40X, 100X, and 200X, respectively). (d) The periodontal ligament (PDL) attached to a thin cementum layer had organized dense fibrous bundles (principle fibers) separated by small areas of vascular connective fibrous tissue. There is no inflammatory cells infiltration. The alveolar bone (black curved up arrow) is compact-dense and contains osteocytes (100X).

Figure 8 shows healthy nonperforated lower rat central incisors, in which the dental pulp exhibits dilated congested blood vessels with multiple layers of palisading columnar odontoblast. The alveolar bone contains osteocyte and PDL attached to thin cementum.

No statistical differences (*P* ≥ 0.05) were observed during the 1st and 4th weeks of the experimental period in the scores of any of the histologic parameters that monitored pulp-dental and periodontium response to the three different types of calcium silicate-based cement (BD, MM-MTA, and ESRRM putty) (Table 3).

## 4. Discussion

Prognosis of root perforation depends on the site, size of the hole, the adjacent periodontal conditions, and sealing materials [4, 34, 35]. The choice of a proper sealing material to seal pathologic or accidental root perforations has a decisive role in the repair of these lesions [36]. This depends on the biocompatibility and bioactivity of the materials promoting the healing process, preserving the vitality of pulp as well as continuing formation of the dentine bridge [25, 37–39], stimulating the repair of the periodontal ligament [40] and regenerating the bone [41].

Clinical studies are essential as they allow inferences about the symptoms and radiographic observation of repaired or unrepaired areas surrounding the periodontal supporting tissues [42, 43]. However, they do not assess tissue responses, which should be the base for accurate material testing, helping to determine the best treatment protocols [1, 2, 44]. Several authors, in assessing the effect of dental materials on pulpal and periodontal tissue responses, have used animal models such as rats [45, 46], although the teeth of dogs and monkeys present size and morphology that facilitate access, visualization, and control of the material to seal perforations [44, 47, 48]. Nonetheless, we found that rats have biological similarities with humans as well as a faster metabolism which reduces the time of research and should, therefore, be considered a practical model for comparing the effects of various materials [45, 46].

In this experiment, designing the lateral perforation in the root of rats' incisors as a model to study dental and paradental hard and soft tissues' response was a pioneering procedure. It adds new knowledge on the biological effects of ESRRM putty in promoting the healing process of experimental perforation related to pulp viability, dentine formation, and bone calcification. Furthermore, it provides simultaneous testing and comparison of the effect of the three calcium silicate-based cements on the inflammatory reaction and regeneration of the pulp-dentine complex and periodontium adjacent to MM-MTA, BD, and ESRRM putty in one model over two different periods. In comparison with previous authors who used the pulp chamber and furcation area of rat molars to assess periodontal and periapical tissue responses to dental materials [45, 47, 49], we showed that the implantation of tested materials in the root of rat incisors is an acceptable and valuable in vivo model. It allows the study of pulpal and periodontium tissue structures and reactions within a single organ and representing the whole life cycle of cellular activity from formation to maturation and repair after the injury as a result of its nature in continuous growth and eruption. Besides, it gives more accessibility with better amounts of bone and PDL to assess the response in comparison to the furcation area or labial-lingual approaches [50, 51].

In this study, we reproduced the biological pulpal response after root perforation. Both ESRRM putty and MM-MTA showed mild inflammation and moderate hyperemia in comparison to BD which showed more congestion and severe inflammatory response with necrosis. The presence of a thin necrotic layer in the pulp of the root treated with BD (score 3) may be due to the released hydroxyl ions that upon hydration increased the pH. This necrotic area protects the underlying vital pulp cells from the material's alkaline pH. Furthermore, it allows the underlying pulp cells to carry out the healing and regeneration functions [52]. However, at the end of the experiment, there were no remarkable differences among the tested materials. The outcome of this study indicates that iatrogenic pulp defects treated with any of the three calcium silicate cements are principally free from serious degenerative tissue reaction after one month of the experiment. These findings are in accordance with the previous investigation that separately found ESRRM putty to be as biocompatible as MTA [53–55], and higher inflammatory response was seen in BD compared with MTA that decreased with time [8, 56].

Otherwise, the existence of the specialized highly differentiated cells (odontoblasts) is crucial during pulp inflammation and trauma. In the best circumstances, at the area of injury, the degenerated cells can be replaced by new odontoblast-like cells that start to form a protective dentine layer [57]. Thus, in the absence of pulpal inflammation, the process of reparative dentinogenesis with complete or partial closing of perforations with dentine-like hard tissue bridges formation was interpreted as a positive reaction to stimulation and as a sign of healing. In this research, the dentine bridge was observed in all samples of ESRRM putty, with better deposition in the BD and MM-MTA groups; however, the differences were not in a statistically significant level. Furthermore, just below the newly formed dentine bridge, the odontoblasts-like palisade cells were also evident in most of the three experimental groups after four weeks, with minor or major structural changes that ranged from mild to complete organization.

The superiority of ESRRM putty over MM-MTA in pulp repair and stimulated dental pulp stem cell proliferation, especially at the beginning of the study, was attributed to its granular topology [58], alkalinity [59], high alkaline phosphatase (ALP) enzyme express [60], and high vascular endothelial growth factor (VEGF) secretion level [61]. Furthermore, the host responses are influenced by the biomaterial properties, and presence of minor amounts of several metal oxides (aluminum, arsenic, beryllium, cadmium, chromium, and irons) in MM-MTA may be another potential draw back [62]. On the other hand, bioceramic products which formed from ESRRM putty contains only trace amount of aluminum, approximately one in thousands of the amount found in MM-MTA [63]. Moreover, presence of bismuth oxide as radiopacifier in MM-MTA does not encourage cell proliferation in cell culture and may increase cytotoxicity compared to the presence of zirconium oxide in BD and tantalum pentoxide in ESRRM putty [11, 55, 64].

Similar results were obtained in the only published in vivo study conducted by Fouda et al. [65]. They assessed the effect of ESRRM putty on the pulp response and concluded that ESRRM putty can induce hard tissue formation to the wall of the exposure site. Moreover, in an in vitro study, Machado et al. [30] reported ESRRM putty and ProRoot MTA had similar rates of mouse dental pulp cells proliferation.

On the other hand, adequate information in line with our results is available both from in vitro and in vivo studies evaluating the effect of BD [23, 56] or different formulations of MTA [66] on the response of the pulp-dentine complex.

Comparing the result of this study with that of other studies in pediatric dentistry, all three tested calcium silicate-based cements can be safely used as MTA in endodontics and pulp treatment [67–69]. As an alternative to MTA, MM-MTA, BD, and ESRRM putty could be suitable due to ease of handling and application, as well as strength and biological effect which is similar to MTA.

Concurrently, our study focused on the effect of ESRRM putty, BD, and MM-MTA on the healing process in the PDL and the surrounding alveolar bone at the site of perforation facing the implanted material. The clinical efficacy of the material depends on its ability to stimulate cell proliferation or survival; this will likely promote the healing process. The results of the histopathologic evaluation showed that after one week, the injured area contained necrotic tissue and disorganized PDL infiltrated by chronic inflammatory cells for all tested groups. Previous in vitro studies had confirmed the good sealing ability of MTA (ProRootM) in repairing large (1 mm) furcation perforations of human molars [70, 71]. Thus, the observed necrotic tissue cannot be attributed to bacterial invasion or the existence of lateral canals as in the case of furcation canals, but is possibly related to the mechanical injury produced during drilling. It is essential to declare that PDL necrosis was not associated with pulpal necrosis in any of the three calcium silicate materials. This is probably due to the abundant number of stem cells and plentiful vascular supply within the pulp of rat incisor as it has an opened apex that has an effect on pulp vitality [50].

Nevertheless, the final outcome after one month affirmed the formation of dense fibrous tissue facing the site of the injured root surface that runs in continuity with the original collagen fibers within the PDL. Besides, some of these fibers mimicked Sharpey's fibers as they were inserted in the cementum-like tissue on the root surface, suggesting the regenerating and organization of the original tissue with continuous eruption in all cases of ESRRM putty without statistical differences in comparison with MM-MTA and BD subgroups.

Regarding the conductive ability of the materials in cortical plate healing after 4 weeks, the present study showed no significant difference among the ESRRM putty, BD, and MM-MTA; however, the ESRRM putty group showed more frequent samples with a complete closure of the hole by a thicker layer of new bone deposition than seen in the MM-MTA and BD groups. This finding is consistent with that of a study conducted by Chen et al. [72]. They observed that six weeks after root-end microsurgery in dogs, ESRRM putty achieved a better PDL and bone-healing response histologically than MTA. ESRRM putty can induce osteoprogenitors to differentiate to mature osteoblasts more than MTA and BD [73, 74]. Recently, Silva et al. [8] stated that although the thickness and area of newly formed mineralized tissue were significantly higher in MTA than BD, BD induced repair by the formation of mineralized tissue sealing, totally or partially, of the furcation perforations in most cases. Also, the osteogenic differentiation potential of human alveolar bone marrow stem cells and hard tissue formation was similar in MM-MTA, ProRoot MTA, and BD [75]. The differentiation of progenitor cells into osteoblast-like cells is essential in the bone healing process, and inducing cell differentiation is required for a biomaterial to be considered a root repair material [76]. The osteodifferentiation of MM-MTA related to increase in alkaline phosphatase activity [77] and the expression of bone morphogenetic protein 2 (BMP2) more than other generations of MTA, and this may be associated with higher concentration of magnesium and sodium [78]. The presence of higher silicon ions in BD stimulates young bone formation by stimulating osteoblasts and promoting mineralization [11].

Future studies are needed to clarify the detailed mechanism of how MM-MTA, BD, and ESRRM putty induced odontogenic proliferation, and osteogenic differentiation of mesenchymal stem cells over an extended treatment period with larger samples is highly recommended.

## 5. Conclusion

Under the limitation of this study, ESRRM putty had early signs of healing process and ended with recognized dentine and bone deposition, followed by MM-MTA and BD. All three calcium silicate-based cements exhibited good biocompatibility to pulp tissue and promoted regeneration of injured PDL and alveolar bone, inducing the proliferation of dental pulp cells and the formation of reparative dentine bridge that increased with time.

## Figures and Tables

**Table 1:** Composition of calcium silicate-based cements used in this study.

Material	Manufacturer	The composition according to the manufacturer	Lot no.
MM-MTA	MicroMega, Besançon, (France)	Powder: tricalcium silicate, dicalcium silicate, tricalcium aluminate, bismuth oxide, calcium sulfate dehydrate, and magnesium oxide. Liquid: calcium carbonate	71708614

ESRRM putty	Brasseler, Savannah, GA, USA	Tricalcium silicate, dicalcium silicate, calcium phosphate monobasic, calcium hydroxide, colloidal silica, and water-free thickening agent	B19585

Biodentine	Septodont, Saint Maur des Fosses, France	Powder: tricalcium silicate (Ca_3_SiO_5_), dicalcium silicate (Ca_2_SiO_4_), calcium carbonate (CaCO_3_), iron oxide (Fe_2_O_3_), and zirconium oxide (ZrO_2_). Liquid: Water (H_2_O) with calcium chloride (CaCl_2_) and soluble polymer (polycarboxylate)	5024200U0

**Table 2:** Evaluation criteria for the histopathological analysis of pulp-dentine complex, PDL, and bone responses after lateral root perforation and mechanical pulp exposure in rats.

0	1	2	3
*Inflammatory cell infiltration of the pulp*			
No necrosis, No hyperemia (no blood vessels and congestion)	No necrosis, normal cellularity, and a minimum amount of blood vessels (<5)	No necrosis, moderate hyperemia, and congestion (>5 blood vessels) with high cellularity (dense fibrous and fibrotic)	Necrosis and severe congestion and hyperemia

*Odontoblast and odontoblastic cell layer*			
Absent	Presence of odontoblastic-like cells only	Partial, incomplete palisading cell pattern	Complete palisading cell pattern

*Dentine bridge formation:*			
No hard tissue deposition	Incomplete (partial or thin irregular D. bridge formation)	Thick, regular, and complete dentine associated with hard tissue formation	

*Bone (calcified bone barrier continuity)*			
No calcified bone barrier formation (deposition)	Limited calcified bone formation extending to no more than one-half of the exposure site	Partial, incomplete calcified bone formation extending to >1/2 of exposure site but not completely closing the exposure site	Complete calcified bone formation

*Periodontal ligament response*			
	Necrosis disorganized PDL with inflammation	Fully organized PDL with no necrosis and inflammation	

**Table 3:** The mean score values of the pulp-dentine complex and periodontium response in the 1st and 4th weeks intervals after filling a lateral root perforation with three types of calcium silicate-based cement.

Parameters	Score range	1^st^ week				4th week			
		BD	MM-MTA	ESRRM putty	*P* value	BD	MM-MTA	ESRRM putty	*P* value
Pulpal response	(0–3)	3 (100%)	2 (100%)	2 (100%)	0.135	0 (100%)	0 (100%)	0.33 (25%)	0.607
Odontoblast	(0–3)	0 (100%)	0 (100%)	0.5 (50%)	0.472	2.5 (50%)	3 (100%)	3 (100%)	0.47
Dentine bridge formation	(0–2)	0 (100%)	1 (25%)	1 (25%)	0.819	1.33 (75%)	1 (100%)	2 (100%)	0.189
Calcific bone barrier	(0–3)	1 (100%)	1 (100%)	1.33 (25%)	0.135	2 (100%)	2 (100%)	3 (100%)	0.082
PDL	(1–2)	1 (100%)	1 (100%)	1.33 (25%)	0.513	1.5 (50%)	2 (100%)	2 (100%)	0.472

## Data Availability

The data used to support the findings of this study are included within the article.
